# *Mycoplasma Bovis* adhesins and their target proteins

**DOI:** 10.3389/fimmu.2022.1016641

**Published:** 2022-10-20

**Authors:** QY. Xu, Q. Pan, Q. Wu, JQ. Xin

**Affiliations:** State Key Laboratory of Veterinary Biotechnology, Harbin Veterinary Research Institute, Chinese Academy of Agricultural Sciences, Harbin, China

**Keywords:** *M. bovis*, adhesin, adhesin binding protein, bovine, vaccine development

## Abstract

Bovine mycoplasmosis is an important infectious disease of cattle caused by *Mycoplasma bovis* (*M. bovis*) which poses a serious threat to the breeding industry. Adhesin is involved in the initial process of *M. bovis* colonization, which is closely related to the infection, cell invasion, immune escape and virulence of this pathogenic microorganism. For the reason that *M. bovis* lacks a cell wall, its adhesin is predominantly located on the surface of the cell membrane. The adhesins of *M. bovis* are usually identified by adhesion and adhesion inhibition analysis, and more than 10 adhesins have been identified so far. These adhesins primarily bind to plasminogen, fibronectin, heparin and amyloid precursor-like protein-2 of host cells. This review aims to concisely summarize the current knowledge regarding the adhesins of *M. bovis* and their target proteins of the host cell. Additionally, the biological characteristics of the adhesin will be briefly analyzed.

## Introduction

*M. bovis*, the causal agent of bovine mycoplasmosis, is a cell wall-free microorganism belonging to the genus *Mycoplasma* and family *Mycoplasmataceae* within the class *Mollicutes*. Bovine mycoplasmosis, currently prevalent all over the world, is one of the severe bovine diseases that cause immeasurable losses to the cattle industry (1). The clinical signs of bovine mycoplasmosis can be manifested as mastitis, pneumonia, arthritis, skin abscess, meningitis, otitis and reproductive tract infection. Furthermore, *M. bovis* is also one of the main pathogens of the bovine respiratory disease complex (BRDC) (2, 3). Cattle are the most vulnerable animal to *M. bovis*, all age groups (preweaning, postweaning, neonate and adult) and all cattle sectors such as beef, milk or rearing could be affected(1). *M. bovis* often causes mixed infection with *Pasteurella multocida (* 4), *Haemophilus somnus (* 5), bovine respiratory syncytial virus (BRSV) (6), bovine herpesvirus 1 (BHV-1) (7), bovine viral diarrhea virus (BVDV)(5), *Histophilus somni (* 8) and parainfluenza virus type 3(6). There is synergy between *M. bovis* and these pathogens during mixed infection.

*M. bovis* was first isolated from a dairy farm with severe mastitis cows in the United States in 1961 (9). Although it has been over 60 years since *M. bovis* was first isolated, the pathogenesis of *M. bovis* is still not very clear. The lack of genomic manipulation methods and expensive animal models cause diagnosis, treatment and vaccine research to stall. It has been confirmed that *M. bovis* infection has consequences on the host immune system, including the production of inflammatory factors or apoptosis of immune cells, and membrane proteins play a critical role in regulating the host defense system (10, 11). In addition, some metabolites of *M. bovis* can also induce host inflammatory reactions, especially following *M. bovis* colonization, it will produce hydrogen peroxide, cause host cell damage in parasitic sites, and mediate more serious inflammatory reactions.

## Adhesion of *M. bovis* to host cells

There is a perception that the cell membrane contact is more conducive to the fusion of Mycoplasma-host cell membrane and the exchange of intracellular components (10). Adhesion is an important step in *M. bovis* infection, the functional proteins (known as adhesins) are closely linked to the pathophysiological process (12, 13). Traditionally, it is generally believed that *M. bovis* mainly parasitizes outside cells, but mounting evidence shows that *M. bovis* can invade host cells, and this mechanism is related to its distribution in host animals and immune escape (14–16). To date, *M. bovis* has been detected in neutrophils, macrophages, bronchial epithelial cells, hepatocytes and kidney cells of infected cattle (17, 18). *In vitro* experimental results also showed that *M. bovis* can invade T cells, B cells, dendritic cells and peripheral blood mononuclear cells such as monocytes and erythrocytes (16). The target proteins of bacterial adhesins are frequently involved in the process of pathogen entry into cells, such as the binding of *Staphylococcus aureus* receptors to fibronectin (FN), which in turn recruits integrins to invade target cells through the endocytic pathway (19). *M. pneumoniae* associates with integrin β1 on the surface of epithelial cells *via* interactions with surface-bound fibronectin and initiates signaling events that stimulate pathogen uptake into clathrin-coated vesicles and caveosomes (20). Some adhesins of *M. bovis* also take FN as the target protein, but whether *M. bovis* invades host cell also adopts the this mechanism remains to be further studied, although recent research shows that clathrin-dependent endocytosis is one of the major pathways by which *M. bovis* invades into synovial cells (14). The invasion of *M. bovis* into different cells may contribute to the spread of pathogens to various colonization sites, which is related to weakening the therapeutic effect of antibiotics and escaping from the immune system killing. *M. bovis* primarily infects cattle, but sheep, goats, pigs, deer and human beings have also been reported to be infected, and *M. bovis* has been isolated from the respiratory tract, udder, joint, heart, brain and other parts of sick cattle (11). The phenomenon of *M. bovis* infecting a variety of animals, tissues and cells suggests that the adhesins of *M. bovis* may be diverse and complex. So far, there are great room for improvement of *M. bovis* vaccine products and the detection methods of *M. bovis*. Furthermore, *M. bovis* is very prone to drug resistance (21), all of these reasons make the prevention, control and elimination of the disease very difficult. Theoretically, the adhesins of mycoplasma are expected to be the candidate vaccine component. In addition, many adhesins have the potential to establish serological detection methods for their excellent immunogenicity.

## Adhesin binding protein

The adhesion of pathogens to specific tissues or cells, a complex process, is the beginning of infection. The target proteins of bacterial adhesins are mainly the components of the host extracellular matrix (ECM), and the protein components of ECM include collagen, elastin, FN, platelet-derived growth factors, laminin and so on (22). For mycoplasma, proteins located on the cell membrane can also bind to ECM components of host cells, such as collagen, laminin, FN, plasminogen (Plg) and glycosaminoglycan heparin, thus mediating mycoplasma colonization or invasion (23). Currently, there are four types of *M. bovis* adhesin binding proteins that have been identified, namely FN, Plg, heparin and amyloid precursor-like protein-2 (APLP-2).

FN is a multifunctional glycoprotein with a high molecular weight, which exists in the forms of soluble dimer in body fluid and insoluble dimer outside cells. The heterodimer is the main form of FN, which is composed of two 230-270 kDa protein chains (24). The dimers are connected by disulfide bonds at the C-end, and each chain is composed of three different types of repeated protein units. These repeating units are called FN domains, which are the basis for FN to perform various functions (25–27). The FN is very conservative in higher animal species. Its key function is to connect the cytoskeleton with the exocellular matrix. FN is one of the crucial binding proteins of bacterial adhesin. At present, more than 100 kinds of bacterial FN target proteins have been identified (25). There is growing evidence that FN and its hydrolysates are related to signal pathways, implying that FN may have other important significance for bacterial infection and invasion in addition to being the target protein of adhesin (27).

Plg is a single chain glycoprotein with a molecular weight of approximately 92 kDa (28). The surface structure of many bacterial pathogens can interact with Plg. These pathogenic microorganisms will recruit Plg to obtain proteolytic activity, promote the invasion of pathogenic bacteria or facilitate their distribution in infected animals (29, 30). Mature Plg mainly exists in two forms: Glu-Plg and Lys-Plg. Glu-Plg is converted into Lys-Plg if the 77 amino-terminal peptides are removed. Both forms of Plg contain seven domains: one activating peptide located in the N-terminal region known as the PAP domain (1 – 77 aa), five kringle domains (Kr1 – 5) and one SP serine protease domain (562 – 791 aa). Plg is an adhesion receptor of a variety of bacteria, fungi and parasites, which is related to the pathogenesis and immune escape of these pathogens (31).

Heparan sulfate (HS) is a widespread form of sulfated glycosaminoglycans, which is present in all types of tissues and cells at extracellular and cellular levels. HS consists of repeating disaccharide units of N-acetylglucosamine (GlcNAc) and hexuronic acid residues. Structurally, the multiple binding activities of HS are closely linked to its extended structural variability. The HS chains are synthesized in the Golgi apparatus by enzymes that initially polymerize alternating N- GlcNAc and glucuronic acid residues. The resulting disaccharide repeats will be variously modified by interdependent reactions, and these modification reactions will not occur uniformly along the chain (32). This mechanism will cause the HS chain to have a wide range of sulfation modes and differential isomerization characteristics, resulting in great diversity, and then provide HS chains with different docking sites for various ligands of polysaccharides. It has been proved that HS proteoglycan is a target protein for the adhesion of a variety of pathogens, which may participate in the internalization process of pathogens and is closely related to the pathogenic mechanism (33). Such as lysated products of *M. hyopneumoniae* P159 binding to haperin facilitated colonized on PK15 cell and may be related to this pathogen internalization (34), the binding of *Candida albicans* to heparin is related to biofilm formation (35), SARS-CoV-2 also applied heparan sulfate as receptor, and several sulfated polysaccharides including heparin show potent anti-SARS-CoV-2 activity (36).

Amyloid precursor-like protein 2 (APLP2) is a member of the amyloid precursor protein family of proteins (APP). APP was found to be evolutionary highly conserved. All APP family members are type 1 integral membrane proteins with a single membrane-spanning domain, a large ectoplasmic N-terminal region and a shorter cytoplasmic C-terminal region (37). The sequences of all APP homologues can be divided into similar domain structures as APP. The ectoplasmic region of APP, which constitutes the major part of the protein, can be divided into the E1 and E2 domains (38). The E1 domain can be further divided into a number of subdomains, including a heparin-binding/growth-factor-like domain (HFBD/GFLD), a copper-binding domain (CuBD) and a zinc-binding domain (ZnBD). The E2 region consists of another HFBD/GFLD and a random coil (RC) region (37). The biological function of APP is still not fully clear. However, it is known that the APP family proteins have redundant and partly overlapping functions (37). The discrete functions of APP including cell adhesion, dendritic outgrowth, axonal transport, synapse formation and synapse modulation, and this diversity of roles is attributed to its numerous proteolytic products (39). Although APP and APLP-2 are related genetically, it has been observed that they are transcriptionally divergent, and there are unique sequence motifs in each gene that suggest specialized, non-overlapping functions (40). APLP-2 has been documented as contributing to pancreatic cancer cell migration, invasiveness, metastasis and copper homeostasis (41). In addition, APLP-2 is an essential component for cell adhesion. There are few reports on APLP-2 as a pathogen receptor, but it has been confirmed that APLP-2 is a target protein for the adhesion of *M. bovis* (42).

## Adhesin of *M. bovis*

So far, 16 proteins have been identified to be involved in the adhesion of *M. bovis* (**Table 1**). These adhesins are basically located on the cell surface. Among these adhesins, seven adhesins have been identified with the clear target protein, three adhesion-related proteins can interact with two different host proteins, and four adhesins are moonlighting proteins with enzyme activities in addition to binding function.

**Table 1:** Summary of *M. bovis* adhesin information.

Adhesin	NCBI Protein ID	No. of aa	MW (kDa)	Signal peptide	Transmembrane region	Target protein	Antigenicity	Subcellular Localization	Moonlight function	Reference
NOX	WP_013954704.1	454	49kDa	No	No	APLP-2 and FN	Unknown	Cytoplasm and cell membrane	Yes	42
MbfN	ADR25360.1	612	96 kDa	Sec/SPII (20-21)	No	FN and heparin	Yes	cell sureface	Unknown	43
FBA	WP_013954544.1	291	34kDa	No	No	FN and Plg	Yes	Cytoplasm and cell membrane	Yes	44/45
TrmFO	AEI89864.1	427	8.548kDa	No	No	FN	Yes	Cytoplasm and cell membrane	Unknown	52
α-Enolase	AEI90157.1	455	49.369kDa	No	No	Plg	Yes	cell sureface	Yes	55
MilA	ADR24994.1	2670	303kDa	Sec/SPI (38-39)	7-29	Haperin	Yes	Whole cell and culture supernatant	Yes	57
P27	MBOV_RS03440	241	27.1kDa	Sec/SPII (24-25)	No	FN	Yes	Cytoplasm and cell membrane	Unknown	58
VpmaX	AEI90145.1	229	35kDa	Sec/SPII (25-26)	7-29	Unknown	Unknown	Cell membrane and whole-cell	Unknown	61
P26 protein	Unknown	Unknown	32kDa	Unkonwn	Unknown	Unknown	Yes	Unknown	Unknown	64
VSPs*	Unknown	Unknown	Unknown	Sec/SPII (23-24/24-25)	No	Unknown	Yes	Cell membrane	Unknown	13/67
Mbov-0503	AFM51859.1	548	59.48kDa	No	7-29	Unknown	Unknown	Cell membrane	Unknown	68
24kDa protein	Unknown	Unknown	24kDa	Unkonwn	Unknown	Unknown	Unknown	Unknown	Unknown	69

*VSPs including VspA, VspB, VspC, VspE and VspF.

### NADH oxidase

NADH oxidase (NOX) is encoded by the NOX gene, and this protein contains 454 amino acids with a molecular weight of 49 kDa (42). NOX has no signal peptide or transmembrane region. The characterization of prokaryotic recombinant NOX protein suggested that it not only had the catalytic activity of oxidase but also functioned as an adhesin. The NOX protein was shown to be distributed in the cytoplasm and cell membrane of *M. bovis*, but it could not be secreted into the culture supernatant. The recombinant NOX protein can bind to EBL cells. Further study found that the protein binds to the membrane protein and cytoplasmic protein of EBL cells in a dose-dependent manner. The reaction with membrane protein is stronger than cytoplasmic protein, and this binding can be specifically blocked by anti-NOX serum. Furthermore, the NOX protein and anti-NOX serum can also block the adhesion of *M. bovis* to EBL cells in a dose-dependent manner. Compared to the parent, a NOX protein-deficient strain showed a decrease in adhesion and H_2_O_2_ producttion. Subsequent experiments confirmed that NOX could specifically adhere to amyloid precursor protein 2 (APLP-2) and FN.

### leucine-rich repeat lipoprotein

James and colleagues found that a leucine-rich repetitive lipoprotein (LRR) has adhesin activity (43). This protein is encoded by the *mbfn* gene of *M. bovis* standard strain PG45. The western blot analysis confirmed that a 48 kDa protein exists in the eight *M. bovis* strains used in their research. In addition to the 48 kDa protein, some strains also have a 70 kDa band. The difference between the two proteins is caused by the loss of 147 amino groups in the C-terminal region. The results of trypsin treatment and hydrophobic protein analysis revealed that this protein was mainly located on the cytomembrane of *M. bovis* and exposed on the cell surface. The protein dot blot assay confirmed that this 48 kDa protein can react with FN. As this was the first proteolytically processed *M. bovis* lipoprotein shown to interact with FN, it was designated as *M. bovis* fibronectin-binding lipoprotein (MbfN). Further experiments confirmed that the binding between MbfN and FN was dose-dependent, saturated adhesion could be achieved at a certain concentration, and anti-MbfN protein polyclonal antibody could specifically block this reaction. Interestingly, the authors found that the protein contains a consensus heparin binding sequence when analyzing it with bioinformatic analysis and then confirmed that MbfN indeed binds to heparin through a dot blot assay. These findings suggest that MbfN is an adhesin with two target proteins, for it can not only react with FN but also bind with heparin. Compared with the original strain, the adhesion ability of the mutant strain with the disrupted open reading frame of MbfN by transposon decreased significantly, and the anti-MbfN antibody could significantly reduce the adhesion of *M. bovis* to MDBK cells.

### Fructose-1,6-diphosphate aldolase

Xiang Gao and Jing Huang identified the adhesin activity of fructose-1,6-diphosphate aldolase (FBA) in 2018 and 2019, respectively (44, 45). FBA is a key enzyme in the process of glycolysis, gluconeogenesis and the Calvin cycle (46). In addition to energy metabolism, FBA also has many biological functions, including acting as Plg binding protein, transcription regulator and participating in host cell adhesion (47–51). FBA protein is composed of 291 amino acids with a molecular weight of 34 kDa. This protein lacks signal peptide or transmembrane region. The FBA is a highly conserved protein of mycoplasma, and the homology of FBA is up to 99% in *M. bovis*. The immunogenicity of FBA was determined by rabbit anti-*M. bovis* serum and it was also shown that the FBA was equally distributed in the cytoplasm and cell membrane. It was confirmed by western blotting and ELISA that the target protein of FBA was Plg. And the results of the adhesion inhibition experiment showed that the FBA antibody could block 34.4% of *M. bovis* adhesion to EBL cells. On this basis, the Jing Huang team further confirmed that the protein can also bind to FN.

### TrmFO

Yongpeng and colleagues discovered that a protein named methylenetetrahydrofolate tRNA - (uracil-5 -) - methyltransferase (TrmFO) has adhesin activity (52). The researchers carried out 150 and 180 generations of wild virulent strain HB0801 *in vitro* at 41°C to prepare a vaccine candidate with a certain protective effect. The genomic analysis found that the down-regulated proteins included NADH oxidase and variable lipoprotein VspX, which was involved in adhesion (53). Using an iTRAQ-based quantitative proteomic analysis, they also found that expression of TrmFO was down-regulated in the attenuated *M. bovis*-150 strain compared to the virulent strain HB0801. The molecular weight of TrmFO is 48.8 kDa and this protein contains 427 amino acids. It does not contain the signal peptide or transmembrane region according to the bioinformatic analysis results. The homology of TrmFO protein among different strains of *M. bovis* was more than 98%. TrmFO was identified as immunogenic by the positive serum of experimentally infected cattle and naturally infected cattle. Through the identification of 8 different isolates of *M. bovis*, it was found that this protein could be expressed in all of the experimental strains. Further immunological tests confirmed that it is a membrane-related protein, which is distributed in both the cytoplasm and cell membrane. The binding protein of TrmFO was identified to be FN by ligand dot blot and ELISA binding assay. The purified TrmFO can adhere to EBL cells, and the rabbit anti-RrmFO antibody significantly reduces the adhesion of purified TrmFO and *M. bovis* to this cell.

### α- Enolase

The α-Enolase of a prokaryote is a highly conserved protein, which is mainly involved in many pathophysiological processes (54). In *M. bovis*, α-Enolase is about 49 kDa in size and contains 454 amino acids. This protein lacks classical protein-sorting signals but contained features typical of Plg binding-site motifs including lysine as the C-terminal residue (FYNIK) and a conserved positively charged lysine-rich internal motif (LYDENSKKY). Zhiqiang and colleagues verified that this protein is present in both the membrane and the soluble cytosolic protein fractions of *M. bovis* cell by western blot. And they also confirmed by ELISA that α-Enolase can indeed bind to Plg in a dose-dependent manner, and this binding can be inhibited by anti-a-enolase serum. Further, they confirmed that the adhesion of *M. bovis* to Plg pretreated EBL cells could be inhibited by rabbit anti-α-Enolase serum (55).

### Mycoplasma immunogenic lipase A

Wawegama and colleagues identified a membrane protein with the potential to establish a detection method for *M. bovis*. This protein is about 226 kDa in size and has lipase activity. The researchers named it Mycoplasma immunogenic lipase A (MilA) (56). Adamu’s team further studied this protein and found that MilA was composed of 3670 amino acids with a molecular weight of 303 kDa. After the protein is expressed, it is hydrolyzed into two fragments of 226 kDa and 50 kDa (57). Sequence analysis showed that MilA contains a glycosaminoglycan motif and multiple copies of a domain of the unknown function (DUF445), and the sequence contains two canonical binding motifs for heparin, “XBBXBX” and “XBBBXXBX”. Trypsin treatment experiments confirmed that the 226 kDa protein was exposed to the surface of mycoplasma, which could bind 1-anilinonaphthalene-8-sulfonic acid, various lipids and heparin. MilA can also bind and hydrolyze ATP, indicating that the protein is likely to be a self-transporter. Antibodies against the carboxyl-terminal of MilA can inhibit the proliferation of *M. bovis in vitro*. The adhesion activity of MilA to heparin shows that the protein plays the role of adhesin, but unfortunately, the author has not conduct classical experiments such as adhesion and adhesion inhibition to further confirm the specific role of this protein in *M. bovis* adhesion.

### p27 protein

It was found that a hypothetical lipoprotein named p27 with adhesin activity by Chen (58). The protein is 27.1 kDa in size and contains 241 amino acids encoded by the gene MBOV_RS03440. As a highly conserved protein, the amino acid homology of p27 between different *M. bovis* strains can reach 100%. It contains a signal peptide and 4 leucine-rich repeat regions (LRRs), but without transmembrane region. The full-length p27 gene could be amplified from the genomes of nine *M. bovis* strains by PCR with primers specific to MBOV_RS03440. And the 27 kDa protein was also specifically recognized by rabbit antiserum to p27 in all tested strains. ELISA assays revealed that recombinant p27 reacted with the sera of cattle naturally and experimentally infected with *M. bovis*. According to the western blot results, the p27 distribute in the whole cells, but most of the p27 molecules were surface exposed. IFA and specific serum blocking assays showed that p27 could directly adhere to EBL cells, and the binding could be specifically blocked by anti-p27 serum. Further experiments confirmed that the target protein of p27 was FN, the interaction of them was direct and specific in a dose-dependent manner.

### VpmaX

The genomic analysis of *M. bovis* Hubei-1 showed that the genome of this strain was missing the VSP gene cluster (59, 60). It was found that there is a gene annotated as VspA in the genome of *M. bovis* strain Hubei-1, but the coding protein of this gene is completely different from the VspA protein of PG45 typical strain. The author designated this gene as VpmaX and studied the adhesion of this gene (61). The VpmaX protein contains 229 amino acids with a molecular mass of approximately 35 kDa. Through bioinformatics analysis, it was found that the protein has a typical prokaryotic signal peptide and two repeat units, namely “KPSEQGSGTNSQQGSG” and “QGSG”, in which the large unit repeated 3 times and the small one repeated 7 times. These structural characteristics are very similar to VSP family proteins. It was confirmed by a immunological method that the protein exclusively located in the cell membrane of *M. bovis*. The VpmaX protein which is expressed in prokaryotes can directly adsorb to EBL cells, and this adhesion to EBL cells can be blocked by anti-VpmaX serum. Interestingly, when the concentration of VpmaX is low, the protein is mainly distributed on the surface of EBL cells, while a higher concentration of VpmaX is used, it can enter the cytoplasm of EBL. Further experiments confirmed that the protein interacted with EBL cells in a dose-dependent manner, and the EBL cell membrane and cytoplasmic components could bind to VpmX in this manner. Unfortunately, while the authors identified VpmaX as an adhesin, they did not identify the binding protein of this adhesin.

### P26 protein

The process of discovering the adhesin P26 protein is anecdotal. As early as 1992, Evelyn and colleagues used the whole bacterial protein of the *M. bovis* J282 strain as an immunogen to screen monoclonal antibodies. Among them, a monoclonal antibody 4F6 that recognizes 26 kDa protein showed fantastic specificity. This monoclonal antibody reacts with all *M. bovis* strains selected in their study (62). In 1993, Sachs found that the adhesion of *M. bovis* to EBL cells can be specifically blocked by monoclonal antibody 4F6. This monoclonal antibody against 26 kDa protein can reduce the adhesion of *M. bovis* strains 120 and 454 to EBL cells by 46% and 70% respectively (63). Under non-blocking conditions, the adhesion ability of 120 strains to EBL cells was higher than that of 454 strains. The results of western blot confirmed that the expression of P26 protein in the strain 120 was higher than that of the strain 454. These results suggested that P26 protein may play an important role in the adhesion of *M. bovis* to EBL cells. In 1995, Sachs purified P26 protein by HPLC and adsorbed EBL cells with P26 protein competing with *M. bovis* strain PG45, 120 and 454. It was found that a 1:100 ratio of P26 protein to the total protein of *M. bovis* could block 20% - 50% of *M. bovis* adsorption (64). Above results provide sufficient evidences that the P26 protein is an adhesin of *M. bovis*. Unfortunately, none of these publications provided the gene sequence or amino acid sequence of the P26 protein, nor identified the target protein of P26 protein.

### Membrane surface variable lipoprotein family proteins

There are membrane surface variable lipoprotein family proteins (VSPs) in *M. bovis*. The members of this family mainly have the following features: (i) an N-terminal portion containing a prokaryotic lipoprotein signal sequence; (ii) a surface-exposed C-terminal region bearing extensive repetitive structures; (iii) high rates of spontaneous non-coordinate phase variation; (iv) high-frequency size variation; and (v) anchorage of these abundantly expressed amphiphilic proteins in the mycoplasma membrane *via* a lipid moiety at an N-terminal cysteine residue (65). Lysnyansky identified that the gene cluster encoding the VSPs in the genome of *M. bovis* standard strain PG45 consists of 15 open reading frames, of which 13 encode VSPs. The research shows that all the amino ends of VSP protein have a conserved prokaryotic signal peptide, with more than 99% homology, and the VSP protein is also anchored on the cell membrane through the amino end (66). Thomas found it by blocking experiments that the monoclonal antibodies 9F1 and 2A8, which recognize VspC and VspF, can inhibit the adhesion of *M. bovis* to bovine bronchial epithelial (BBE) cells, and the monoclonal antibody 1E5 also has a certain blocking ability. These results indirectly suggested that VspC and VspF may be an adhesin (13). Sachse and colleagues conducted immunogenicity and adhesion studies on VspA, VspB, VspE and VspF of *M. bovis (* 67). When the sequence analysis of the above VSPs, they identified that each sequence contained repetitive sequences. Artificially synthesized repetitive sequences or antibodies against these specific sequences was applied to conduct inhibit adhesion experiments of *M. bovis*, and the investigations showed that VspA, VspB, VspE and VspF reduced the adhesion of *M. bovis*, and monoclonal antibodies against VspA, VspB and VspF also inhibited the adhesion of *M. bovis* to host cells. The above results indicated that VspA, VspB, VspC, VspE and VspF are *M. bovis* adhesins. It is worth noting that some monoclonal antibodies which inhibit the adhesion of *M. bovis* to EBL cells could not inhibit the adhesion to BBE cells (13). This result suggests that the adhesins of *M. bovis* against distinct host cells may be different. It is also a pity that neither of the two studies identified the target proteins of these VSPs adhesins.

### Mbov_0503 coding protein

Through the screening of transposon mutant strains, Zhu and colleagues identified an adhesin encoded by the Mbov_0503 gene. This adhesin of *M. bovis* contains 548 amino acids. They carried out a prokaryotic expression of the adhesin and obtained a soluble protein with a molecular weight of 59.4 kDa (68). In that research, they first established the transposon mutation library for M. *bovis* HB0801 and screened 9 strains with decreased adhesion in MDBK cells and EBL cells, among which mbov_0503 protein mutant strain has the most stable characteristics, and supplement of mbov_0503 can restore the adhesion ability of mutant. Further analysis found that mbov_0503 protein on the surface of *M. bovis*. The results of laser scanning confocal microscopy and ELISA confirmed that Mbov_0503 protein could directly adhere to the surface of EBL cells and bind to EBL cell membrane proteins in a dose-dependent manner. The researchers found that the adhesin could only partially affect the adhesion of *M. bovis* to host cells and that the mutation had no effect on the proliferation of the mutant, but the deletion of Mbov_0503 resulted in a significant decrease in the ability of the mutant to cross the cell barrier. Although not verified in animal experiments, this adhesion is likely to be closely related to the pathogenicity of mycoplasma. Furthermore, the ligand for Mbov_0503 was not identified in this study.

### 24 kDa proteins

As mentioned above, Thomas demonstrated that *M. bovis* can adhere to bovine tracheal epithelial cells (BBE) (13). Their follow-up study found that the adhesion ability of *M. bovis* 2610 strains decreased significantly after passage *in vitro*. The results of 2D electrophoresis analysis showed that the expression of a protein with a size of about 24 kDa was significantly reduced in the high passage strain. The sequence of this protein was obtained by LC-MS/MS analysis, but it does not match any of the known sequences in the *M. bovis* database by BLAST software. Trypsin digestion experiments showed that the 24 kDa protein was a membrane protein, and serum or monoclonal antibody against this protein could reduce the adhesion of 2610P7 (passage 7 *in vitro*) strain to BBE cells. According to the above observations, this protein with a size of 24 kDa is an adhesin (69).

## Adhesin processing

Post-translational processing of adhesin is a relatively common phenomenon, including signal peptide excision and post-translational hydrolysis. So far, this phenomenon has been found in many pathogens, such as *M. pneumoniae*, *M. hyopneumoniae*, *streptococcus* bacteria, some parasites, etc. The processing phenomenon of classical adhesin p97 and P102 family proteins of *M. hyopneumoniae* is universal. In *M. hyopneumoniae*, it was not only that multiple adhesins have such post-translational processing, but also adhesin-related proteins (70). Post-translational processing is essential for the colonization, adhesion and pathogenicity of *M. hyopneumoniae* (71, 72). And the adhesin protein of *M. hyopneumoniae* often shows multiple cleavage sites, resulting in different cleavage products showing different adhesion characteristics. For example, the p123j protein of *M. hyopneumoniae* has cleavage events at multiple sites, and the product of this protein can bind to diverse host cell surface components (73). Another mycoplasma that has fully demonstrated the phenomenon of adhesin processing is *M. pneumoniae*. Studies have shown that almost half (317; 46%) of ORFs derived from *M. pneumoniae* strain M129 are post-translationally modified (74). *M. pneumoniae* needs a complex attachment organelle to achieve adhesion and mobility functions, and its main components include adhesins P1 and P30. The major adhesins, P1 and P30, are localized to the tip of the attachment organelle by the surface-accessible cleavage fragments P90 and P40 derived from *Mpn*142 (75). In bacteria, the treatment of adhesion proteins is also crucial. For example, *Streptococcus* adhesion protein AbpA needs the treatment of SrtB to have the ability to adhere to salivary amylase. The absence of SrtB brings about AbpA to be released into the culture supernatant, and *Streptococcus* loses the ability to bind salivary amylase (76). In addition, this post-translation processing of adhesin is also identified in parasites (77). In *M. bovis* adhesins, post-translational processing has also been confirmed (43, 57), but the research on this phenomenon is not in-depth. The treatment of adhesin may be closely related to the exposure of adhesin binding sites, immune escape, invasion and toxicity differences. Considering this, this part of the work needs to be increased in the study of *M. bovis* adhesins in the future.

## Biological functions of adhesins and their target proteins

**Figure 1:**
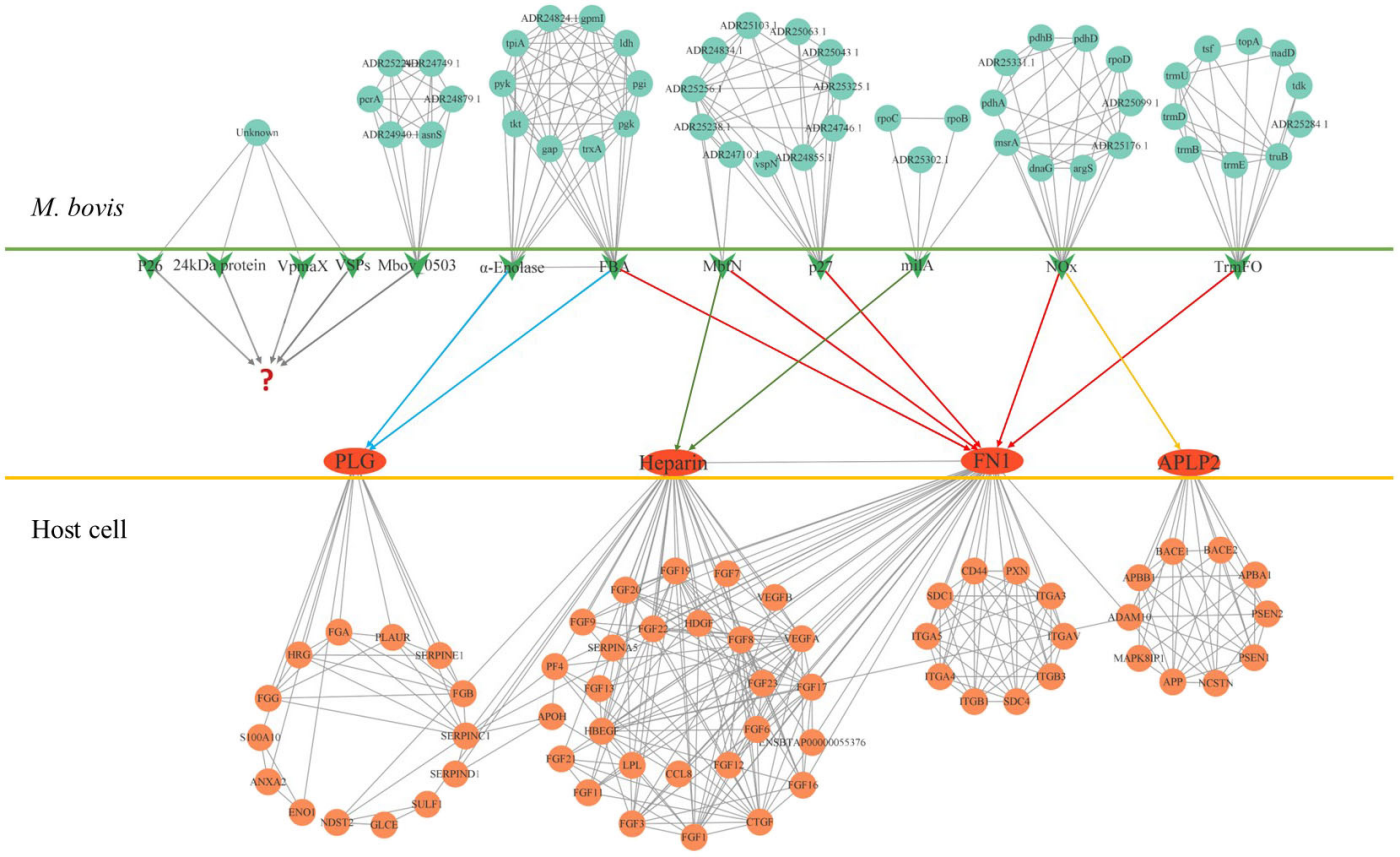
The protein interaction network of *M. bovis* adhesin and adhesin-binding protein. The green arrows in the figure represent adhesins, and the red ovals represent adhesin-binding proteins. Cyan circles represent proteins that interact with adhesin in *M. bovis*, light red circles represent proteins that interact with adhesin-binding proteins of the host, and question marks represent unidentified adhesin-binding proteins. The relationship of the adhesin to the corresponding binding protein is indicated by the line segment with the arrow.

**Figure 2:**
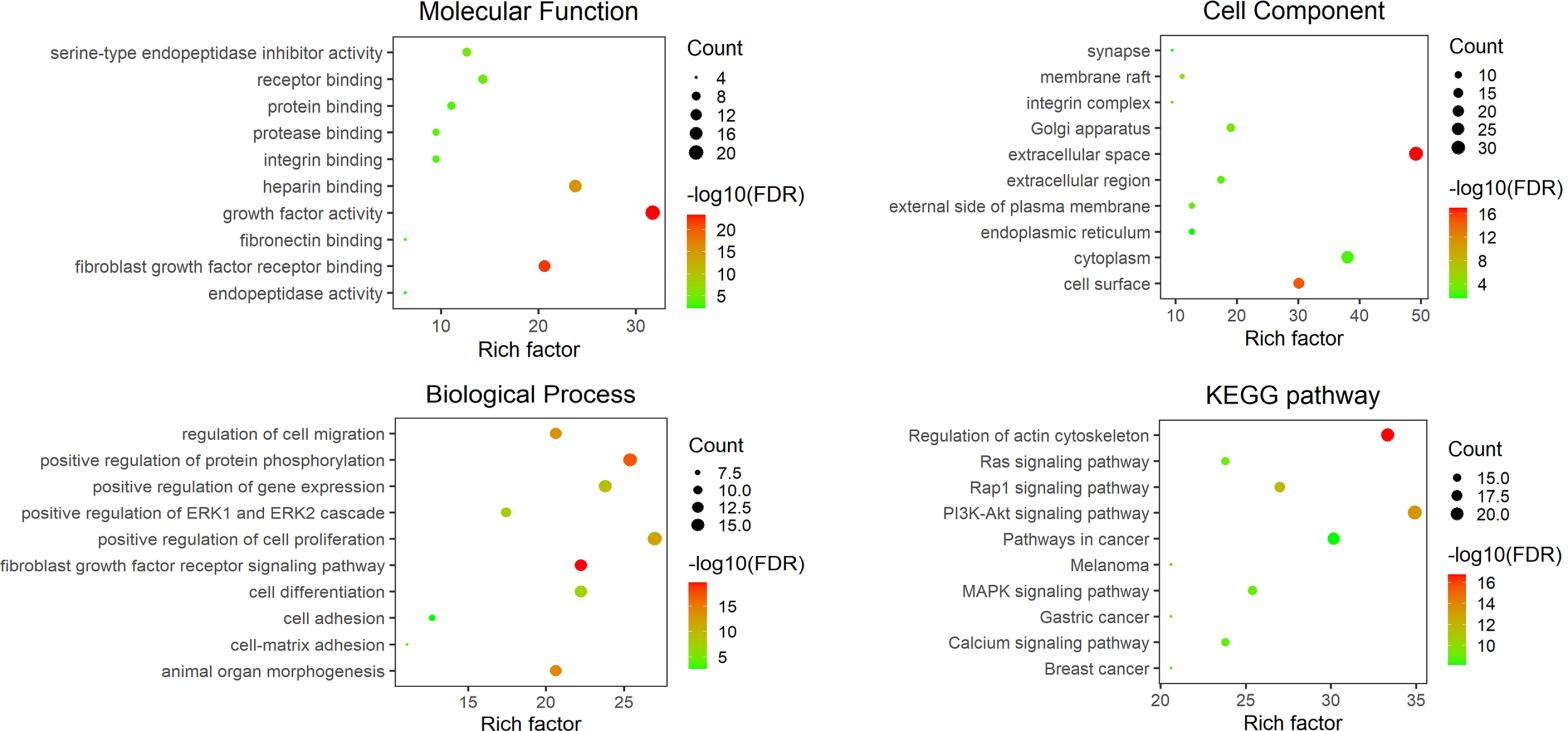
The GO and KEGG analysis results of related components of the host adhesin binding protein interaction network. The figure shows the top ten entries with an FDR of less than 0.05. The ordinate represents the GO annotation entry or KEGG pathway name, and the abscissa is a rich factor. The number of enrichment genes is associated with bubble size, and the statistical significance is associated with bubble color.

Limited by the information obtained from existing studies, we know little about the role of *M. bovis* adhesin’s effect on host cells and itself in infection. Based on the reported adhesins of *M. bovis*, host binding proteins and STRING database, all the interacting proteins was shown in **Figure 1**. Among these adhesins, the relative proteins of FBA and α-Enolase are highly coincident, and the interaction proteins of MbfN also belong to the interaction protein network of p27, which may mean that these adhesins have the same or similar physiological effects on *M. bovis* in the process of infection, and this physiological effect may be more important for the survival of *M. bovis* in specific host tissues. Unfortunately, we were still unable to obtain the interaction protein information of P26, VSPs, VpmaX and 24 kDa protein. To adhesin binding proteins, the proteins that interact with FN1 were divided into two groups, one of which was highly coincident with heparin-related proteins. Further, according to the database KOBAS, we found that the adhesin-related pathways maybe associated with biological activities such as biosynthesis and energy metabolism. *M. bovis* infection cause a series of biological reactions in the host cells, which may promote the pathogen colonization or adverse effects to the host. Based on the DAVID database information, the adhesin-binding proteins were not only related to various cancer signaling pathways proteins, but also involved in cell adhesion, connectivity, apoptosis, inflammatory responses and many other processes (**Figure 2**).

Although it is not easy to define the biological function of adhesin on *M. bovis* itself, this part of work is indeed an important aspect of *M. bovis* adhesin research. At the same time, the effect of adhesin on the host also needs further verification, especially the protein interaction network and signal pathway related to pathogenicity. At present, the study is very rare to independently verify the effect of *M. bovis* adhesin on host cells, but such research is of great significance for advancing the research of *M. bovis* pathogenicity and vaccines.

## Application potential of adhesin in vaccine development

Because the antibiotic treatment is not working as expected (78), an effective vaccine has become one of the desirous means expected to prevent and control the disease. In recent years, magnitude work has focused on the terms of *M. bovis* vaccine research. Scientists have made attempts in inactivated vaccines, attenuated vaccines and subunit vaccines. Unfortunately, they have not achieved ideal results (79). As mentioned above, most adhesins of *M. bovis* are membrane surface proteins, with excellent antigenicity, and play critical roles in infection, pathogen proliferation and host damage. Therefore, these proteins have great potential in the *M. bovis* vaccine developing. The problems faced by the development of the *M. bovis* subunit vaccine are mainly the following aspects: 1. Some candidate proteins may be protective but lack conservatism. Such as VSPs, are considered to be potential vaccine candidate proteins. However, the antigenic variation in these lipoproteins may make the vaccines ineffective in the long run (67); 2. Some candidate components not only failed to provide protection but will aggravate clinical signs, such as higher lesions in lung injury (80); 3. It is insufficient available selection space for high-quality vaccine candidate protein. Although a variety of vaccines containing single protein or multi-protein have been exploited, the expected effect has not been achieved. Researchers have tried a variety of proteins, including glyceraldehyde-3-phosphate dehydrogenase alone as a vaccine (80, 81), PdhA, PepA, Tuf, P48, P81, OppA, LppA, PepQ, O256 and DeoB combination as a vaccine (82), and *M. bovis* membrane fractions and cell extracts, etc. (83). In fact, given the role of *M. bovis* adhesin in its infection, this kind of protein may be more suitable for vaccine research and development. When combined with reverse vaccinology and structural biology, it may have a better prospect. For example, the α-enolase of *M. bovis* can be expressed on the surface of the pathogen and combined with plasminogen. These characteristics are very similar to the α-enolase of *Streptococcus suis*, which shows good protection in mice and zebrafish models (84, 85). It is believed that with the help of emerging technologies and the deepening of adhesin research, a new situation will be opened in the field of vaccine research.

## Conclusion

Although some progress has been made in the study of bovine adhesins, there is still much room for improvement in this field. The research of *M. bovis* adhesin is relatively backward in the whole field of mycoplasma. The shortcomings are mainly as follows: 1. The technics of adhesin identification need to be further improved. In addition to the methods mentioned in this review, other technologies such as IP/Co-IP, mass spectrometry, proteomics, and bioinformatics can also be used to identify adhesin; 2. The research on the mechanism of the function realization of adhesins is not deep enough, especially on the post-translational processing; 3. Does *M. bovis* adhesin require the involvement of chaperones, and if so what are these chaperones and how do they work? These issues also need to be clarified; 4. The difference of *M. bovis* adhesin in different host cells needs further study, which is of great significance to further explain the infection of *M. bovis* to multiple organs and tissues; 5. In addition to adhering to host cells, the question of what effect does bovine mycoplasma adhesin on host cells has not been well clarified. In this review, based on the identified *M. bovis* adhesins, adhesin-binding proteins and online database, we discussed the possible biological effects of the adhesins on the pathogen and host cells. These possible biological effects have not been verified, but are inferred from available information. Strictly, the support of the existing literature and arguments in this part of the discussion is not sufficient. On one hand, this discussion hopes to attract the attention of relevant researchers, and on the other hand, it also hopes to provide limited background knowledge for such research.

Many excellent researchers and teams have made outstanding contributions to the research of *M. bovis* adhesins, and more than 10 adhesins have been identified. Adhesins have special significance in the process of *M. bovis* infection, and some adhesins are very well-conservative and have excellent immunogenicity. Many adhesins have a variety of biological activities, which may be related to the virulence of *M. bovis*. Therefore, the study of *M. bovis* adhesin may be of great benefit to the progress of serological detection methods, vaccines, and pathogenesis of *M. bovis*.

## Author contributions

QX, QP and QW writing-original draft; QX and JX designed and finally revised the article. All authors contributed to the article and approved the submitted version.

## Funding

This work was supported by National Natural Science Foundation of China (Grant No. 31872468).

## Conflict of interest

The authors declare that the research was conducted in the absence of any commercial or financial relationships that could be construed as a potential conflict of interest.

## Publisher’s note

All claims expressed in this article are solely those of the authors and do not necessarily represent those of their affiliated organizations, or those of the publisher, the editors and the reviewers. Any product that may be evaluated in this article, or claim that may be made by its manufacturer, is not guaranteed or endorsed by the publisher.

## References

[1] Dudek K Nicholas RAJ Szacawa E Bednarek D. Mycoplasma bovis infections-occurrence, diagnosis and control. Pathogens (2020) 9:640. doi: 10.3390/pathogens9080640 PMC7459460 32781697

[2] Oliveira TES Pelaquim IF Flores EF Massi RP Valdiviezo MJJ Pretto-Giordano LG. Mycoplasma bovis and viral agents associated with the development of bovine respiratory disease in adult dairy cows. Transbound Emerg Dis (2020) 67:82–93. doi: 10.1111/tbed.13223 31232526 PMC7228412

[3] Maunsell FP Woolums AR Francoz D Rosenbusch RF Step DL Wilson DJ. Mycoplasma bovis infections in cattle. J Vet Intern Med (2011) 25:772–83. doi: 10.1111/j.1939-1676.2011.0750.x 21745245

[4] Nicholas RA Ayling RD. Mycoplasma bovis: disease, diagnosis, and control. Res Vet Sci (2003) 74:105–12. doi: 10.1016/S0034-5288(02)00155-8 12589733

[5] Shahriar FM Clark EG Janzen E West K Wobeser G. Coinfection with bovine viral diarrhea virus and mycoplasma bovis in feedlot cattle with chronic pneumonia. Can veterin J = La Rev veterin Can (2002) 43:863–8. PMC339759 12497963

[6] Mehinagic K Pilo P Vidondo B Stokar-Regenscheit N. Coinfection of Swiss cattle with bovine parainfluenza virus 3 and mycoplasma bovis at acute and chronic stages of bovine respiratory disease complex. J Vet Diagn Invest (2019) 31:674–80. doi: 10.1177/1040638719861686 PMC6727125 31246162

[7] Prysliak T van der Merwe J Lawman Z Wilson D Townsend H van Drunen Littel-van den Hurk S. Respiratory disease caused by mycoplasma bovis is enhanced by exposure to bovine herpes virus 1 (BHV-1) but not to bovine viral diarrhea virus (BVDV) type 2. Can veterin J = La Rev veterin Can (2011) 52:1195–202. PMC3196011 22547839

[8] Booker CW Abutarbush SM Morley PS Jim GK Pittman TJ Schunicht OC. Microbiological and histopathological findings in cases of fatal bovine respiratory disease of feedlot cattle in Western Canada. Can veterin J = La Rev veterin Can (2008) 49:473–81. PMC2359492 18512458

[9] Hale HH Helmboldt CF Plastridge WN Stula EF. Bovine mastitis caused by a mycoplasma species. Cornell veterin (1962) 52:582–91. 13952069

[10] Bürki S Frey J Pilo P. Virulence, persistence and dissemination of mycoplasma bovis. Vet Microbiol (2015) 179:15–22. doi: 10.1016/j.vetmic.2015.02.024 25824130

[11] Calcutt MJ Lysnyansky I Sachse K Fox LK Nicholas RAJ Ayling RD. Gap analysis of mycoplasma bovis disease, diagnosis and control: An aid to identify future development requirements. Transbound Emerg Dis (2018) 65 Suppl 1:91–109. doi: 10.1111/tbed.12860 29582590

[12] Thomas A Sachse K Dizier I Grajetzki C Farnir F Mainil JG. Adherence to various host cell lines of mycoplasma bovis strains differing in pathogenic and cultural features. Vet Microbiol (2003) 91:101–13. doi: 10.1016/S0378-1135(02)00303-6 12458160

[13] Thomas A Sachse K Farnir F Dizier I Mainil J Linden A. Adherence of mycoplasma bovis to bovine bronchial epithelial cells. Microb Pathog (2003) 34:141–8. doi: 10.1016/S0882-4010(03)00003-2 12631475

[14] Nishi K Gondaira S Fujiki J Katagata M Sawada C Eguchi A. Invasion of mycoplasma bovis into bovine synovial cells utilizing the clathrin-dependent endocytosis pathway. Vet Microbiol (2021) 253:108956. doi: 10.1016/j.vetmic.2020.108956 33373880

[15] Bürki S Gaschen V Stoffel MH Stojiljkovic A Frey J Kuehni-Boghenbor K. Invasion and persistence of mycoplasma bovis in embryonic calf turbinate cells. Vet Res (2015) 46:53. doi: 10.1186/s13567-015-0194-z 25976415 PMC4432498

[16] van der Merwe J Prysliak T Perez-Casal J. Invasion of bovine peripheral blood mononuclear cells and erythrocytes by mycoplasma bovis. Infect Immun (2010) 78:4570–8. doi: 10.1128/IAI.00707-10 PMC2976333 20713619

[17] Maeda T Shibahara T Kimura K Wada Y Sato K Imada Y. Mycoplasma bovis-associated suppurative otitis media and pneumonia in bull calves. J Comp Pathol (2003) 129:100–10. doi: 10.1016/S0021-9975(03)00009-4 12921715

[18] Kleinschmidt S Spergser J Rosengarten R Hewicker-Trautwein M. Long-term survival of mycoplasma bovis in necrotic lesions and in phagocytic cells as demonstrated by transmission and immunogold electron microscopy in lung tissue from experimentally infected calves. Vet Microbiol (2013) 162:949–53. doi: 10.1016/j.vetmic.2012.11.039 23294861

[19] Foster TJ. The remarkably multifunctional fibronectin binding proteins of staphylococcus aureus. Eur J Clin Microbiol Infect Dis Off Publ Eur Soc Clin Microbiol (2016) 35:1923–31. doi: 10.1007/s10096-016-2763-0 27604831

[20] Raymond BBA Turnbull L Jenkins C Madhkoor R Schleicher I Uphoff CC. Mycoplasma hyopneumoniae resides intracellularly within porcine epithelial cells. Sci Rep (2018) 8:17697. doi: 10.1038/s41598-018-36054-3 30523267 PMC6283846

[21] Lysnyansky I Ayling RD. Mycoplasma bovis: Mechanisms of resistance and trends in antimicrobial susceptibility. Front Microbiol (2016) 7:595. doi: 10.3389/fmicb.2016.00595 27199926 PMC4846652

[22] Patel S Mathivanan N Goyal A. Bacterial adhesins, the pathogenic weapons to trick host defense arsenal. Biomed pharmacother = Biomed pharmacother (2017) 93:763–71. doi: 10.1016/j.biopha.2017.06.102 28709130

[23] Li J Wang J Shao J Li Y Yu Y Shao G. The variable lipoprotein family participates in the interaction of mycoplasma hyorhinis with host extracellular matrix and plasminogen. Vet Microbiol (2022) 265:109310. doi: 10.1016/j.vetmic.2021.109310 34954543

[24] Dalton CJ Lemmon CA. Fibronectin: Molecular structure, fibrillar structure and mechanochemical signaling. Cells (2021) 10:2443. doi: 10.3390/cells10092443 34572092 PMC8471655

[25] Henderson B Nair S Pallas J Williams MA. Fibronectin: a multidomain host adhesin targeted by bacterial fibronectin-binding proteins. FEMS Microbiol Rev (2011) 35:147–200. doi: 10.1111/j.1574-6976.2010.00243.x 20695902

[26] Takahashi S Leiss M Moser M Ohashi T Kitao T Heckmann D. The RGD motif in fibronectin is essential for development but dispensable for fibril assembly. J Cell Biol (2007) 178:167–78. doi: 10.1083/jcb.200703021 PMC2064432 17591922

[27] Dallo SF Kannan TR Blaylock MW Baseman JB. Elongation factor tu and E1 beta subunit of pyruvate dehydrogenase complex act as fibronectin binding proteins in mycoplasma pneumoniae. Mol Microbiol (2002) 46:1041–51. doi: 10.1046/j.1365-2958.2002.03207.x 12421310

[28] Vassalli JD Sappino AP Belin D. The plasminogen activator/plasmin system. J Clin Invest (1991) 88:1067–72. doi: 10.1172/JCI115405 PMC295552 1833420

[29] Yavlovich A Katzenell A Tarshis M Higazi AA Rottem S. Mycoplasma fermentans binds to and invades HeLa cells: involvement of plasminogen and urokinase. Infect Immun (2004) 72:5004–11. doi: 10.1128/IAI.72.9.5004-5011.2004 PMC517474 15321992

[30] Lottenberg R. A novel approach to explore the role of plasminogen in bacterial pathogenesis. Trends Microbiol (1997) 5:466–7. doi: 10.1016/S0966-842X(97)01171-2 9447656

[31] Ayón-Núñez DA Fragoso G Bobes RJ Laclette JP. Plasminogen-binding proteins as an evasion mechanism of the host's innate immunity in infectious diseases. Biosci Rep (2018) 38:BSR20180705. doi: 10.1042/BSR20180705 30166455 PMC6167496

[32] Vivès RR Seffouh A Lortat-Jacob H. Post-synthetic regulation of HS structure: The yin and yang of the sulfs in cancer. Front Oncol (2014) 3:331. doi: 10.3389/fonc.2013.00331 24459635 PMC3890690

[33] García B Fernández-Vega I García-Suárez O Castañón S Quirós LMJ. The role of heparan sulfate proteoglycans in bacterial infections. J Med Micob Diagnosis (2014) 3:1. doi: 10.4172/2161-0703.1000157

[34] Burnett TA Dinkla K Rohde M Chhatwal GS Uphoff C Srivastava M. P159 is a proteolytically processed, surface adhesin of mycoplasma hyopneumoniae: defined domains of P159 bind heparin and promote adherence to eukaryote cells. Mol Microbiol (2006) 60:669–86. doi: 10.1111/j.1365-2958.2006.05139.x 16629669

[35] Green JV Orsborn KI Zhang M Tan QK Greis KD Porollo A. Heparin-binding motifs and biofilm formation by candida albicans. J Infect Dis (2013) 208:1695–704. doi: 10.1093/infdis/jit391 PMC4038792 23904295

[36] Tandon R Sharp JS Zhang F Pomin VH Ashpole NM Mitra D. Effective inhibition of SARS-CoV-2 entry by heparin and enoxaparin derivatives. J Virol (2021) 95:e01987–20. doi: 10.1128/JVI.01987-20 33173010 PMC7925120

[37] Jacobsen KT Iverfeldt K. Amyloid precursor protein and its homologues: a family of proteolysis-dependent receptors. Cell Mol Life Sci (2009) 66:2299–318. doi: 10.1007/s00018-009-0020-8 PMC11115575 19333550

[38] Gralle M Ferreira ST. Structure and functions of the human amyloid precursor protein: the whole is more than the sum of its parts. Prog Neurobiol (2007) 82:11–32. doi: 10.1016/j.pneurobio.2007.02.001 17428603

[39] Midthune B Tyan SH Walsh JJ Sarsoza F Eggert S Hof PR. Deletion of the amyloid precursor-like protein 2 (APLP2) does not affect hippocampal neuron morphology or function. Mol Cell Neurosci (2012) 49:448–55. doi: 10.1016/j.mcn.2012.02.001 PMC3348437 22353605

[40] Pandey P Sliker B Peters HL Tuli A Herskovitz J Smits K. Amyloid precursor protein and amyloid precursor-like protein 2 in cancer. Oncotarget (2016) 7:19430–44. doi: 10.18632/oncotarget.7103 PMC4991393 26840089

[41] Sliker BH Goetz BT Peters HL Poelaert BJ Borgstahl GEO Solheim JC. Beta 2-microglobulin regulates amyloid precursor-like protein 2 expression and the migration of pancreatic cancer cells. Cancer Biol Ther (2019) 20:931–40. doi: 10.1080/15384047.2019.1580414 PMC6606018 30810435

[42] Zhao G Zhang H Chen X Zhu X Guo Y He C. Mycoplasma bovis NADH oxidase functions as both a NADH oxidizing and O(2) reducing enzyme and an adhesin. Sci Rep (2017) 7:44. doi: 10.1038/s41598-017-00121-y 28246386 PMC5427908

[43] Adamu JY Mitiku F Hartley CA Sansom FM Marenda MS Markham PF. Mycoplasma bovis mbfN encodes a novel LRR lipoprotein that undergoes proteolytic processing and binds host extracellular matrix components. J Bacteriol (2020) 203:e00154–20. doi: 10.1128/JB.00154-20 33077633 PMC7950404

[44] Huang J Zhu H Wang J Guo Y Zhi Y Wei H. Fructose-1,6-bisphosphate aldolase is involved in mycoplasma bovis colonization as a fibronectin-binding adhesin. Res Vet Sci (2019) 124:70–8. doi: 10.1016/j.rvsc.2019.02.010 30852357

[45] Gao X Bao S Xing X Fu X Zhang Y Xue H. Fructose-1,6-bisphosphate aldolase of mycoplasma bovis is a plasminogen-binding adhesin. Microb Pathog (2018) 124:230–7. doi: 10.1016/j.micpath.2018.08.032 30142464

[46] Raymond BB Djordjevic S. Exploitation of plasmin(ogen) by bacterial pathogens of veterinary significance. Vet Microbiol (2015) 178:1–13. doi: 10.1016/j.vetmic.2015.04.008 25937317

[47] Chaves EG Weber SS Báo SN Pereira LA Bailão AM Borges CL. Analysis of paracoccidioides secreted proteins reveals fructose 1,6-bisphosphate aldolase as a plasminogen-binding protein. BMC Microbiol (2015) 15:53. doi: 10.1186/s12866-015-0393-9 25888027 PMC4357084

[48] Ziveri J Tros F Guerrera IC Chhuon C Audry M Dupuis M. The metabolic enzyme fructose-1,6-bisphosphate aldolase acts as a transcriptional regulator in pathogenic francisella. Nat Commun (2017) 8:853. doi: 10.1038/s41467-017-00889-7 29021545 PMC5636795

[49] Rodaki A Young T Brown AJ. Effects of depleting the essential central metabolic enzyme fructose-1,6-bisphosphate aldolase on the growth and viability of candida albicans: implications for antifungal drug target discovery. Eukary Cell (2006) 5:1371–7. doi: 10.1128/EC.00115-06 PMC1539134 16896220

[50] Tunio SA Oldfield NJ Berry A Ala'Aldeen DA Wooldridge KG Turner DP. The moonlighting protein fructose-1, 6-bisphosphate aldolase of neisseria meningitidis: surface localization and role in host cell adhesion. Mol Microbiol (2010) 76:605–15. doi: 10.1111/j.1365-2958.2010.07098.x 20199602

[51] Han X Zhu X Hong Z Wei L Ren Y Wan F. Structure-based rational design of novel inhibitors against fructose-1,6-Bisphosphate aldolase from candida albicans. J Chem Inf model (2017) 57:1426–38. doi: 10.1021/acs.jcim.6b00763 28475320

[52] Guo Y Zhu H Wang J Huang J Khan FA Zhang J. TrmFO, a fibronectin-binding adhesin of mycoplasma bovis. Int J Mol Sci (2017) 18:1732. doi: 10.3390/ijms18081732 PMC5578122 28792486

[53] Zhang R Han X Chen Y Mustafa R Qi J Chen X. Attenuated mycoplasma bovis strains provide protection against virulent infection in calves. Vaccine (2014) 32:3107–14. doi: 10.1016/j.vaccine.2013.12.004 24462404

[54] Pancholi V. Multifunctional alpha-enolase: its role in diseases. Cell Mol Life Sci (2001) 58:902–20. doi: 10.1007/PL00000910 PMC11337373 11497239

[55] Song Z Li Y Liu Y Xin J Zou X Sun W. Alpha-enolase, an adhesion-related factor of mycoplasma bovis. PloS One (2012) 7:e38836. doi: 10.1371/journal.pone.0038836 22719960 PMC3374825

[56] Wawegama NK Browning GF Kanci A Marenda MS Markham PF. Development of a recombinant protein-based enzyme-linked immunosorbent assay for diagnosis of mycoplasma bovis infection in cattle. Clin Vaccine Immunol CVI (2014) 21:196–202. doi: 10.1128/CVI.00670-13 24334686 PMC3910936

[57] Adamu JY Wawegama NK Kanci Condello A Marenda MS Markham PF Browning GF. Mycoplasma bovis membrane protein MilA is a multifunctional lipase with novel lipid and glycosaminoglycan binding activity. Infect Immun (2020) 88:e00945–19. doi: 10.1128/IAI.00945-19 32253247 PMC7240078

[58] Chen X Huang J Zhu H Guo Y Khan FA Menghwar H. P27 (MBOV_RS03440) is a novel fibronectin binding adhesin of mycoplasma bovis. Int J Med Microbiol IJMM (2018) 308:848–57. doi: 10.1016/j.ijmm.2018.07.006 30076003

[59] Qi J Guo A Cui P Chen Y Mustafa R Ba X. Comparative geno-plasticity analysis of mycoplasma bovis HB0801 (Chinese isolate). PloS One (2012) 7:e38239. doi: 10.1371/journal.pone.0038239 22693604 PMC3365025

[60] Li Y Zheng H Liu Y Jiang Y Xin J Chen W. The complete genome sequence of mycoplasma bovis strain hubei-1. PloS One (2011) 6:e20999. doi: 10.1371/journal.pone.0020999 21731639 PMC3120828

[61] Zou X Li Y Wang Y Zhou Y Liu Y Xin J. Molecular cloning and characterization of a surface-localized adhesion protein in mycoplasma bovis hubei-1 strain. PloS One (2013) 8:e69644. doi: 10.1371/journal.pone.0069644 23936063 PMC3720590

[62] Berthold E Heller M Pfützner H Leirer R Sachse K. Preparation and characterization of monoclonal antibodies against mycoplasma bovis. Zentralblatt fur Veterin Reihe B J veterin Med Ser B (1992) 39:353–61. doi: 10.1111/j.1439-0450.1992.tb01180.x 1519412

[63] Sachse K Pfützner H Heller M Hänel I. Inhibition of mycoplasma bovis cytadherence by a monoclonal antibody and various carbohydrate substances. Vet Microbiol (1993) 36:307–16. doi: 10.1016/0378-1135(93)90097-Q 7505986

[64] Sachse K Grajetzki C Rosengarten R Hänel I Heller M Pfützner H. Mechanisms and factors involved in mycoplasma bovis adhesion to host cells. Zentralblatt fur Bakteriol Int J Med Microbiol (1996) 284:80–92. doi: 10.1016/S0934-8840(96)80157-5 8837372

[65] Beier T Hotzel H Lysnyansky I Grajetzki C Heller M Rabeling B. Intraspecies polymorphism of vsp genes and expression profiles of variable surface protein antigens (Vsps) in field isolates of mycoplasma bovis. Vet Microbiol (1998) 63:189–203. doi: 10.1016/S0378-1135(98)00238-7 9850998

[66] Lysnyansky I Sachse K Rosenbusch R Levisohn S Yogev D. The vsp locus of mycoplasma bovis: gene organization and structural features. J bacteriol (1999) 181:5734–41. doi: 10.1128/JB.181.18.5734-5741.1999 PMC94094 10482515

[67] Sachse K Helbig JH Lysnyansky I Grajetzki C Müller W Jacobs E. Epitope mapping of immunogenic and adhesive structures in repetitive domains of mycoplasma bovis variable surface lipoproteins. Infect Immun (2000) 68:680–7. doi: 10.1128/IAI.68.2.680-687.2000 PMC97192 10639433

[68] Zhu X Dong Y Baranowski E Li X Zhao G Hao Z. Mbov_0503 encodes a novel cytoadhesin that facilitates mycoplasma bovis interaction with tight junctions. Microorganisms (2020) 8:164. doi: 10.3390/microorganisms8020164 PMC7074692 31979335

[69] Thomas A Leprince P Dizier I Ball H Gevaert K Van Damme J. Identification by two-dimensional electrophoresis of a new adhesin expressed by a low-passaged strain of mycoplasma bovis. Res Microbiol (2005) 156:713–8. doi: 10.1016/j.resmic.2005.02.008 15950126

[70] Machado L Paes JA Souza Dos Santos P Ferreira HB. Evidences of differential endoproteolytic processing on the surfaces of mycoplasma hyopneumoniae and mycoplasma flocculare. Microb Pathog (2020) 140:103958. doi: 10.1016/j.micpath.2019.103958 31899326

[71] Djordjevic SP Cordwell SJ Djordjevic MA Wilton J Minion FC. Proteolytic processing of the mycoplasma hyopneumoniae cilium adhesin. Infect Immun (2004) 72:2791–802. doi: 10.1128/IAI.72.5.2791-2802.2004 PMC387856 15102789

[72] Paes JA Machado L Dos Anjos Leal FM De Moraes SN Moura H Barr JR. Comparative proteomics of two mycoplasma hyopneumoniae strains and mycoplasma flocculare identified potential porcine enzootic pneumonia determinants. Virulence (2018) 9:1230–46. doi: 10.1080/21505594.2018.1499379 PMC6104684 30027802

[73] Raymond BB Jenkins C Seymour LM Tacchi JL Widjaja M Jarocki VM. Proteolytic processing of the cilium adhesin MHJ_0194 (P123J ) in mycoplasma hyopneumoniae generates a functionally diverse array of cleavage fragments that bind multiple host molecules. Cell Microbiol (2015) 17:425–44. doi: 10.1111/cmi.12377 25293691

[74] Berry IJ Widjaja M Jarocki VM Steele JR Padula MP Djordjevic SP. Protein cleavage influences surface protein presentation in mycoplasma pneumoniae. Sci Rep (2021) 11:6743. doi: 10.1038/s41598-021-86217-y 33762641 PMC7990945

[75] Widjaja M Berry IJ Pont EJ Padula MP Djordjevic SP. P40 and P90 from Mpn142 are targets of multiple processing events on the surface of mycoplasma pneumoniae. Proteomes (2015) 3:512–37. doi: 10.3390/proteomes3040512 PMC5217387 28248283

[76] Liang X Liu B Zhu F Scannapieco FA Haase EM Matthews S. A distinct sortase SrtB anchors and processes a streptococcal adhesin AbpA with a novel structural property. Sci Rep (2016) 6:30966. doi: 10.1038/srep30966 27492581 PMC4974636

[77] Baker RP Wijetilaka R Urban S. Two plasmodium rhomboid proteases preferentially cleave different adhesins implicated in all invasive stages of malaria. PloS Pathog (2006) 2:e113. doi: 10.1371/journal.ppat.0020113 17040128 PMC1599764

[78] Andrés-Lasheras S Jelinski M Zaheer R McAllister TA. Bovine respiratory disease: Conventional to culture-independent approaches to studying antimicrobial resistance in north America. Antibiotics (2022) 11:487. doi: 10.3390/antibiotics11040487 35453238 PMC9025279

[79] Perez-Casal J Prysliak T Maina T Suleman M Jimbo S. Status of the development of a vaccine against mycoplasma bovis. Vaccine (2017) 35:2902–7. doi: 10.1016/j.vaccine.2017.03.095 28433326

[80] Prysliak T van der Merwe J Perez-Casal J. Vaccination with recombinant mycoplasma bovis GAPDH results in a strong humoral immune response but does not protect feedlot cattle from an experimental challenge with m. bovis Microb Pathog (2013) 55:1–8. doi: 10.1016/j.micpath.2012.12.001 23246808

[81] van der Merwe J Prysliak T Gerdts V Perez-Casal J. Protein chimeras containing the mycoplasma bovis GAPDH protein and bovine host-defence peptides retain the properties of the individual components. Microb Pathog (2011) 50:269–77. doi: 10.1016/j.micpath.2010.11.008 21296650

[82] Prysliak T Perez-Casal J. Immune responses to mycoplasma bovis proteins formulated with different adjuvants. Can J Microbiol (2016) 62:492–504. doi: 10.1139/cjm-2015-0762 27105454

[83] Mulongo M Prysliak T Perez-Casal J. Vaccination of feedlot cattle with extracts and membrane fractions from two mycoplasma bovis isolates results in strong humoral immune responses but does not protect against an experimental challenge. Vaccine (2013) 31:1406–12. doi: 10.1016/j.vaccine.2012.12.055 23340004

[84] Membrebe JD Yoon NK Hong M Lee J Lee H Park K. Protective efficacy of streptococcus iniae derived enolase against streptococcal infection in a zebrafish model. Veterin Immunol immunopathol (2016) 170:25–9. doi: 10.1016/j.vetimm.2016.01.004 26872628

[85] Wang J Wang K Chen D Geng Y Huang X He Y. Cloning and characterization of surface-localized α-enolase of streptococcus iniae, an effective protective antigen in mice. Int J Mol Sci (2015) 16:14490–510. doi: 10.3390/ijms160714490 PMC4519854 26121302

